# Graphene-Based Biosensor for Early Detection of Iron Deficiency

**DOI:** 10.3390/s20133688

**Published:** 2020-07-01

**Authors:** Oluwadamilola Oshin, Dmitry Kireev, Hanna Hlukhova, Francis Idachaba, Deji Akinwande, Aderemi Atayero

**Affiliations:** 1 Electrical and Information Engineering Department, Covenant University, Ota 112233, Nigeria; francis.idachaba@covenantuniversity.edu.ng (F.I.); atayero@covenantuniversity.edu.ng (A.A.); 2 Department of Electrical and Computer Engineering, The University of Texas at Austin, Austin, TX 78712, USA; kirdmitry@gmail.com (D.K.); deji@ece.utexas.edu (D.A.); 3 Microelectronics Research Center, The University of Texas at Austin, Austin, TX 78758, USA; 4 Institute of Complex Systems (ICS-8), Forschungszentrum Juelich, 52428 Jülich, Germany; gaoa1994@gmail.com

**Keywords:** biosensor, early detection, ferritin, graphene, GFET, iron deficiency, nanotechnology, non-invasive

## Abstract

Iron deficiency (ID) is the most prevalent and severe nutritional disorder globally and is the leading cause of iron deficiency anemia (IDA). IDA often progresses subtly symptomatic in children, whereas prolonged deficiency may permanently impair development. Early detection and frequent screening are, therefore, essential to avoid the consequences of IDA. In order to reduce the production cost and complexities involved in building advanced ID sensors, the devices were fabricated using a home-built patterning procedure that was developed and used for this work instead of lithography, which allows for fast prototyping of dimensions. In this article, we report the development of graphene-based field-effect transistors (GFETs) functionalized with anti-ferritin antibodies through a linker molecule (1-pyrenebutanoic acid, succinimidyl ester), to facilitate specific conjugation with ferritin antigen. The resulting biosensors feature an unprecedented ferritin detection limit of 10 fM, indicating a tremendous potential for non-invasive (e.g., saliva) ferritin detection.

## 1. Introduction

Nutrition during the early years of life has a preeminent influence on the quality of health of an individual in their lifetime [1,2,3]. Specifically, micronutrients provide the essential building blocks for brain development, healthy growth, and a robust immune system [1,4,5,6,7]. The top three micronutrients of global health relevance are iodine, iron, and vitamin A, whereas iron deficiency is the most common nutritional disorder worldwide [8,9].

Iron deficiency (ID) refers to a condition of significantly low concentration of healthy red blood cells in the body due to the correspondingly low amount of iron [10,11]. The core function of iron in the body is oxygen transport in the blood. Iron deficiency, if not diagnosed and treated at the early stage, will lead to iron deficiency anemia (IDA). Although every age group is vulnerable, it is more prevalent in women and children [8,12]. However, it is often impossible to recognize ID in children until it degenerates to IDA. At that point, symptoms such as pale skin, frequent infections, fatigue/lethargy, pica, and poor appetite become apparent. ID impairs the cognitive development of children from infancy through to adolescence and is associated with increased morbidity rates [13,14]. It is, therefore, imperative to be able to promptly detect iron deficiencies in children, so that intervention programs are timely and better targeted.

Although, iron status is best assessed by a combination of indicators, ferritin is established as the major iron-storage molecule; its production increases in cells as iron supplies increase. The serum ferritin level is, therefore, the most specific biochemical test that correlates with relative total body iron stores; hence, it is the most widely used iron status indicator [8]. However, since ferritin is an acute-phase reactant protein, its concentration is elevated in the presence of infection or inflammation. A child under five years of age is said to be iron-deficient if their serum ferritin level is <12 µg/L, while the threshold is <15 µg/L for children over five years old but rises to <30 µg/L in the presence of an infection [15]. Hence, ferritin tests should be taken very seriously when the results are abnormally low compared to when the measure is normal [10]. 

Investigating iron deficiency involves a continuous process of recording and assessing iron status in an individual to identify a drop in the indicator levels. Therefore, non-invasiveness becomes necessary, especially when children are involved. The use of saliva presents a non-invasive approach. Saliva is known to contain every information present in the blood but in significantly smaller quantities. Some research works demonstrated the use of saliva for micronutrient testing [16,17]. Moreover, research went into determining salivary ferritin concentrations in humans, as well as correlating serum (or plasma) and salivary ferritin concentrations, as presented in Table 1.

From Table 1, it is clear that the lowest literature-reported iron-deficient salivary ferritin concentration is 0.186 µg/L, which is significantly lower than the 12 µg/L iron-deficient serum ferritin concentration level. The significantly low levels of ferritin in human saliva make it impossible to use the current micronutrient biosensors presented in the literature (presented in Table 2).

The detection mechanisms of all sensors reported in Table 2, except the silicon nanowire type [29], are all optoelectronic. The biosensor detection range of the lateral flow immunoassay (LFIA)-based sensor [27] is 3–556 µg/L in buffer and 5.78–888 µg/L in serum ferritin standard. However, the sensitivity and specificity reported are based on a detection limit of 18 µg/L. Ferritin concentrations lower than this cut-off resulted in a degradation of the sensitivity and specificity of the biosensor. The silicon nanowire detection mechanism is a departure from the rest in its utilization of a nano-field-effect transistor (FET). This presented the significant advantage of a lower detection limit as compared with others. From an extensive literature search, it was observed that there are significantly few studies on the detection of ferritin concentration using field-effect biosensors. Their method attained a ferritin detection limit down to 50 pg/mL using a horn-like polycrystalline-silicon nanowire (SiNW) FET. Even though their fabrication method is acclaimed to be simpler, the synthesis of silicon nanowires is generally non-trivial and expensive [30,31]. On the other hand, graphene synthesis is simple; graphene is widely commercially available and inexpensive. Moreover, unlike SiNWs, the two-dimensional (2D) planar surface structure of graphene facilitates ease of functionalization. Graphene fabrication and transfer to the substrate are also significantly simple compared to the procedure for SiNWs [32]. 

Since the first exfoliation of a single atomic layer of graphene in 2004 by Geim and Novoselov [33], of all other nanomaterials, it is known to be the most promising nanostructured material suitable for biosensing, under intense research for over a decade [34,35]. 

In this research work, we developed an FET biosensor using monolayer graphene as the conducting channel. We functionalized the channel using anti-ferritin antibodies to selectively capture the ferritin protein antigen, with a limit of detection about 10 fM. It is noteworthy that this performance was attained despite using our low-cost and straightforward shadow mask patterning procedure to derive the source and drain electrodes of the graphene-based FETs (GFETs), rather than the standard (ultraviolet (UV) or e-beam) lithography process [36]. This work is the first report of ferritin detection using graphene. It also offers the lowest ferritin detection limit obtainable by any reported sensor. This work demonstrates the enormous potential of using a GFET for non-invasive early detection of iron deficiency.

## 2. Materials and Methods

### 2.1. Materials

Graphene on 25-µm-thick copper foil (Gr/Cu) synthesized through chemical vapor deposition (CVD) was purchased from Chongqing Graphene Technology Co., Ltd. (also known as Chongqing Moxi Technology). The following materials were ordered from Millipore Sigma (formerly Sigma-Aldrich): ferritin, anti-ferritin antibody, dimethylformamide (DMF), Tween-20, ethanolamine (ETA), and ~150 mM phosphate-buffered saline (1× PBS, pH 7.4 at 25 °C). Here, 1.5 mM PBS (0.01× PBS) was prepared by diluting 1× PBS appropriately with de-ionized water. Furthermore,1-pyrenebutanoic acid, succinimidyl ester (PASE) was purchased from Thermofisher Scientific. 

### 2.2. GFET Fabrication

**Figure 1:**
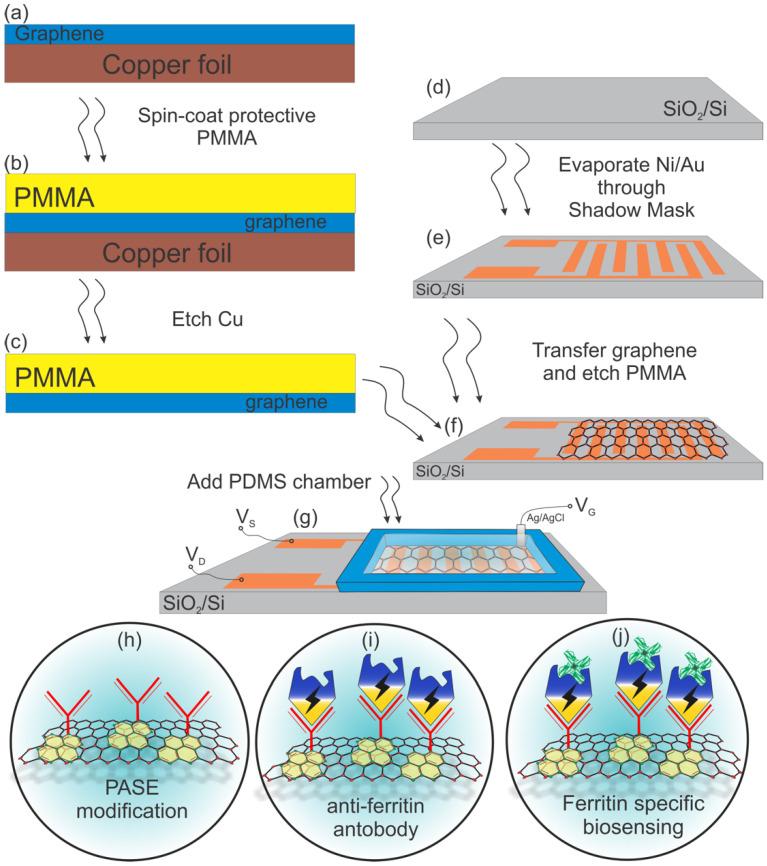
(**a**–**f**) Graphene-based field-effect transistor (GFET) biosensor fabrication process; (**g**) the schematic of the final GFET-based biosensor with a polydimethylsiloxane (PDMS) well on top to secure electrolyte; (**h**–**j**) further graphene functionalization with pyrenebutanoic acid, succinimidyl ester (PASE), an anti-ferritin antibody and the final step of ferritin-specific biosensing.

A 285-nm-thick SiO_2_ on Si wafer was used as a substrate. The source and the drain of the transistor used in this work were patterned according to an interdigitated electrode (IDE). The IDE-structured transistors were fabricated using a shadow mask to pattern the electrodes on top of the SiO_2_/Si substrate. The masks were fabricated via a simple yet robust technology, which allows for fast prototyping of desirable patterns at a fraction of time and cost, and which utilizes a commercially available, off-the-shelf tool, Silhouette Cameo, capable of providing resolution down to 200 um [37]. A detailed account of this process was reported elsewhere [36]. Although this is a more straightforward approach to patterning in contrast with lithography, there is a limit on the sizes obtainable due to the resolution of the mechanical cutting machine. Each SiO_2_/Si wafer yielded 28 transistors based on the pre-set dimensions (see Figure S1, Supplementary Materials). Since an IDE structure was used, the overall length of the channel was set to 1 mm, and the width was set to 68.8 mm, yielding a ~69 W/L ratio. Using the CHA e-beam-assisted evaporator, a thin layer each of Ni (10 nm) and Au (90 nm) was deposited. Nickel was deposited first to serve as an adhesion layer, while gold was the metal contact serving as the source and drain for the transistor. The purchased Gr/Cu was cut into desired sizes and stuck onto dummy silicon wafers. A protective polymer (poly(methyl methacrylate) (PMMA)) was drop-casted onto the Gr/Cu and spin-coated for even distribution. The resulting PMMA/Gr/Cu was then annealed at 150 °C for 5 min. This was thereafter transferred onto the etchant (0.1 M ammonium persulfate) to remove the underlying copper foil, leaving PMMA/Gr on top of the solution. The PMMA/Gr was then triple-washed with deionized (DI) water, followed by a careful transfer of a PMMA/Gr sheet onto each IDE-structured transistor to bridge the source and drain electrodes. After the transfer, PMMA/graphene was left to slowly dry out for 12 h at room temperature, followed by 5 min of 150 °C annealing in order to re-flow the PMMA and improve graphene-substrate adhesion. The devices were then left for 24 h in acetone in order to remove the protective PMMA layer, then washed with IPA, and dried with an oxygen gun. Polydimethylsiloxane (PDMS) chambers were then molded and attached to each chip to form an exposed well above the graphene sensing area, thereby creating the means for liquid-based measurements. The GFET fabrication process is summarized in Figure 1a–g.

### 2.3. GFET Functionalization

Transforming a GFET into a specific biosensor requires immobilization of the necessary biomolecules as seen in Figure 1h–j. To immobilize the biomolecules, a bi-functional linker molecule) 1-pyrenebutanoic acid, succinimidyl ester (PASE) was firstly introduced to the graphene surface. The graphene channel area was incubated in the solution of 10 mM PASE in DMF for 2 h at room temperature. The aromatic pyrene groups of PASE bind very strongly but non-covalently to the graphene surface via π–π stacking, leaving the succinimidyl ester group at the other end (see Figure 1h). Next, a 1 mg/mL solution of anti-ferritin antibody in 1× PBS was introduced to the GFETs and incubated for 12 h at 4 °C. In effect, the succinimidyl ester groups of PASE react covalently with the amino groups of the antibody, forming a stable amide bond (see Figure 1i). After incubation, the GFETs were washed thoroughly with 1× PBS and DI water, then dried with compressed nitrogen. The graphene surface was then washed with 0.05% Tween-20 in 0.01× PBS to passivate the unbound graphene surface or physically trapped biomolecules. Finally, an additional blocking step of 100 mM ETA in 0.01× PBS was applied to the graphene surface for 1 h at room temperature to deactivate any unreacted succinimidyl ester groups of the PASE that may remain on the surface, followed by thorough washing in 0.01× PBS and DI water, and drying with compressed nitrogen (see Figure 1j). The GFET functionalization process is summarized in Figure S2 (Supplementary Materials).

After functionalization, the target analyte, ferritin, was prepared in 0.01× PBS to obtain the desired concentrations.

### 2.4. GFET Characterization

Prior to functionalization, we took the Raman spectra of the graphene on our IDE FET substrate via the Renishaw inVia Raman microscope, using the blue excitation laser wavelength of 442 nm and 4 mW power on the sample to verify the graphene quality and number of layers. The GFETs were electrically characterized at room temperature prior to functionalization, after antibody immobilization and after applying the blocking buffer (Tween-20 and ETA). All measurements were based on a liquid-gated FET set-up, as shown in Figure S3 (Supplementary Materials). We used 0.01× PBS as the electrolyte buffer solution, and a Keithley B2902A Source Measure Unit (SMU) coupled to a Wentworth Labs probe station. Ag/AgCl pellet electrodes (E-206, Science Products) were used as gate reference electrodes, and they were carefully washed between experiments in order to avoid any cross-contamination. The immobilization processes were characterized by monitoring the drain current changes for a drain-source voltage (V_DS_) of 0.2 V while sweeping the gate voltage (V_GS_) from −0.5 to 0.5 V. The sensor performance was determined by monitoring the drain current changes per time for a given drain-source voltage and gate voltage, as the GFET was exposed to the different concentrations of ferritin. The time-trace recordings were performed while keeping both V_DS_ and V_GS_ constant at a certain operational point. The point was set to be V_DS_ = 0.1 V and V_GS_ = 0.05 V to make sure there were no excessive currents through the graphene.

## 3. Results and Discussion

As shown in Figure S4 (Supplementary Materials), the used graphene was a high-quality monolayer, as verified by the I_2D_/I_G_ ratio >2 [38]. The Raman spectrum also revealed a minimal D peak at 1350 cm^−1^, showing very low defect density. The quality of this graphene facilitated consistent GFET transport properties and confirmed the high fabrication yield of >99% as specified by the manufacturer.

**Figure 2:**
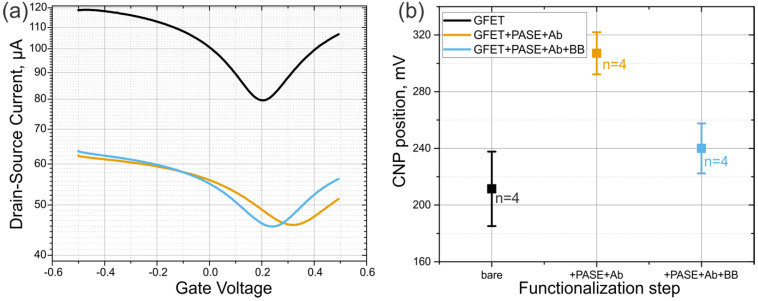
(**a**) Transfer curves of a GFET upon functionalization process. Black, orange, and blue lines represent the bare GFET, the GFET functionalized with PASE and antibodies, and the functionalized GFET after passivation with blocking buffer (BB). (**b**) Statistics of the CNP shift upon the same functionalization steps from *n* = 4 similar devices.

Characterizations were based on transfer curves obtained by plots of drain current versus the gate voltage during stages of fabrication and functionalization of the GFET. The transfer curve obtained by characterizing the bare GFETs immediately after fabrication showed that the GFETs had an average and positive-valued Dirac voltage (Vdirac) of 211 ± 60.4 mV (Figure 2, black line). This monolayer graphene Dirac point corresponds to the charge neutrality point (with a mean value of Vcnp = 211 mV) or the point of least conductivity/ maximum resistance. Pristine monolayer graphene (non-doped) has a Vcnp of 0 V; graphene is said to be p-doped when Vcnp is positive and n-doped when Vcnp is negative. Specific anti-ferritin antigen binding was achieved by functionalizing the GFET with an anti-ferritin antibody using PASE as the linker to the graphene surface; this yielded an average Vdirac of 307 ± 33.54 mV (Figure 2, yellow curve). Usage of the anti-ferritin specific antibody gives us an immense advantage of building a single type of molecule specific biosensor that will be sensitive to ferritin only. Next, a blocking buffer comprising a wash step with 0.05% Tween-20 was applied to remove unbound biomolecules from the graphene surface as much as possible, before incubating the functionalized channel with ETA to block the remaining unreacted N –hydroxy succinimidyl (NHS) ester linkers on the channel surface. Specificity of the biosensor was also assured by means of a previously tested method of additional biosensor passivation performed with ethanolamine and Tween-20 [39,40]. This last functionalization step yielded an average Vdirac of 240 ± 40 mV (Figure 2, blue line). The decrease in Vdirac was likely due to the removal of weakly bound antibody probes and the nullifying of remnant NHS–ester linker molecules. The standard deviations (SD) of the provided charge neutrality point (CNP) values come from the analysis of multiple (*n* = 4) different GFET chips that were fabricated in a similar manner. Figure S5 (Supplementary Materials) shows the shift of the I–V curve upon functionalization steps for another device that was not used for time-trace recording of ferritin, but to see the steady-state shift in the current response.

### 3.1. Ferritin Detection

The liquid-gated FET (LG-FET) measurement set-up is the primary measurement configuration for biosensors, where the “liquid” is the sample containing the analyte to be detected or quantified. In this LG-FET set-up, the gate voltage that triggers the modulations in the device is applied to a reference electrode through the liquid to the graphene channel. As this potential is applied, the ELECTRICAL DOUBLE LAYER (EDL) with a capacitance value of C_EDL_ is formed just above the graphene channel. In effect, the C_EDL_ in series with the air-gap capacitance due to graphene’s hydrophobicity and the inherent quantum capacitance of graphene produce the total gate capacitance of the GFET. Therefore, a significant advantage of this set-up is the low operating voltage required for the device, typically within 1 V. The thickness of the EDL is a function of the Debye length (λ_D_) as seen in Equation (1).

When antigens bind to their antibodies immobilized on the FET surface, a change in surface charge is induced at the binding site. For the changes to be effectively captured, the binding site must be within the Debye length, defined by Equation (2) [41]. Therefore, changes that occur outside this length are subject to electrostatic charge screening.

(1) CEDL=ε0εrλD

(2) λD=0.304M[nm]

where $\varepsilon_{0}$ is the permittivity of free space, $\varepsilon_{r}$ is the relative permittivity of the dielectric formed between the graphene surface and the liquid, and M (molarity) is the ionic strength of the sample (liquid).

From Equation (2), it is evident that a higher molarity results in a shorter Debye length. This concept is of great concern because most biological interactions take place within high-ionic-strength solutions (e.g., 1× PBS ionic strength = ~150 mM). In effect, an attempt to sense these interactions electronically using FET-based sensors is severely impeded by the consequentially short Debye length (0.7 nm for 1× PBS). Therefore, although the binding efficiency of ferritin and its antibody is high due to its large molecular size [42], to ensure this binding is detected by the GFET biosensor, 0.01× PBS (M = 1.5 mM, λ_D_ = 7.3 nm) was used as the electrolyte to carry out the measurements.

It is also clear from Figure S2 (Supplementary Materials) that the functionalization process incurs some height on the graphene surface that eats into the Debye length. However, the literature highlights that the incurred height from the sensor surface after a flat-on-orientation immobilization of the antibodies is typically about 4 nm [29,43]. Therefore, even for macromolecular antigens like ferritin, using 0.01× PBS will give room for detection of the antigen–antibody binding since the binding site will be within the Debye length of ~7.3 nm.

**Figure 3:**
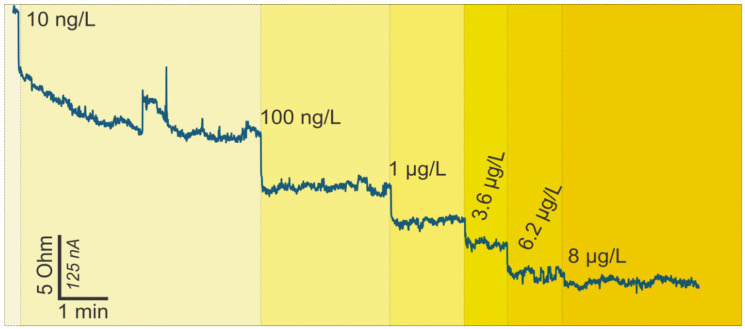
Change in resistance versus time readings for the GFET ferritin biosensor on the addition of ferritin-free buffer (phosphate-buffered saline (PBS)) and increasing ferritin concentration.

For a p-type GFET device, the number of holes is greater than the number of electrons; hence, on the application of the gate voltage, decreased conductivity results. On the other hand, when the GFET is n-type, the application of the gate voltage leads to increased conductivity. However, the immobilization and the binding of charged target biomolecules to receptors on the channel yield specific channel modulation effects. For a p-type device, when a negatively charged biomolecule binds to the receptors on the graphene channel, holes accrue in the channel, leading to increased drain-source current [44]. This binding corresponds to a negative gating potential of the graphene channel and, hence, the reduced carrier density of graphene [45]. On the contrary, when a positively charged biomolecule binds to the receptors on the graphene channel, reduced drain-source current results [46]. Ferritin is a negatively charged molecule with a weight of 474 kDa [47,48,49]; therefore, with a GFET operated in hole-conduction mode, it is expected that the drain-source current increases (resistance decreases) as the antigen is immobilized on the device. Monitoring of current change is carried out at a certain working potential (0.05 V in this case), and the shift of current is a typical response of biomolecule attachment [40,50,51,52,53]. This expected trend can be observed in Figure 3. This figure also represents the points where the highlighted ferritin concentrations pipetted onto the chip resulted in the depicted electrical changes. In this experiment, the ferritin was added onto the chip with initially clean PBS solution. Using simple calculations and a set of four stock ferritin solutions, we gradually increased the concentration of ferritin in the sensing bath, without cleaning or removing the liquid in between. This allowed us to record the gradual change in the response due to the increase in ferritin concentration in a single experiment.

Concerning the detection limit and range of the GFET biosensor, we started pipetting the ferritin antigen onto the chip from the smallest concentration of 10 ng/L, consequently increasing the ferritin concentration up to 8 µg/L. The initial concentration resulted in a significant rise in drain current, which suggests that the smallest analyte concentrations detectable by the developed GFETs are actually lower than 10 ng/L. Notably, the changes in drain current upon ferritin immobilization occurred within less than 10 s of pipetting the protein onto the GFETs, portraying real-time detection.

### 3.2. Ferritin Binding Kinetics

We consider A (antibody) and F (ferritin) to be two interacting bio-objects which can form a bound product, AF, and we let C_A_, C_F_, and C_AF_ be their concentration in M (molarity). The time-dependent rate equation for the formation of the product C_AF_ is

(3) $$\frac{dc_{AF}}{dt}=k_{on}c_{A}c_{F}$$

where the forward reaction rate constant is $k_{on}$, and the reverse reaction rate constant is $k_{off}$.

(4) $$\frac{dc_{AF}}{dt}=-k_{off}c_{A}{}_{F}$$

In equilibrium, the sum of all time-dependent derivatives is zero, which, in fundamental interpretation, obeys the law-of-mass-action equation in solution [54].

(5) $$K_{a}=\frac{1}{K_{D}}=\frac{k_{on}}{k_{off}}=\frac{c_{AF}}{c_{A}\times c_{F}}$$

$$c_{AF}=K_{a}\times c_{A}\times c_{F}$$

The strength of the interaction between A (antibody) and F (ferritin) can be linked to the affinity constant Ka via the concentration of bound ferritin molecules to the concentration of antibodies. However, it is also necessary to consider the dissociation constant K_D_, because it can be compared to the reactant ferritin concentrations. In solution, the total concentration of bound antibody–ferritin complex (C_AF_) depends on the concentration of both antibody and ferritin for biosensors with active surface areas where the law-of-mass-action applies [55].

Immobilized antibodies on the biosensor surface are fixed and, thus, the number of captured ferritin molecules will not change. To have an ideal experiment, the number of ferritin antigens should be in large excess with respect to the number of immobilized antibodies, such that the effective total concentration does not change when ferritin antigens adsorb from the solution to the surface.

**Figure 4:**
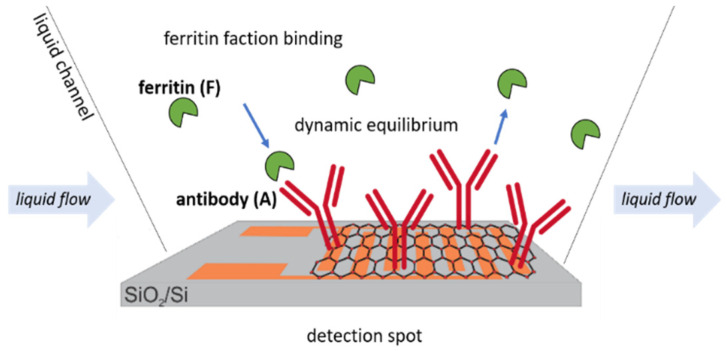
Schematic representation of the dynamic equilibrium of ferritin antigens to immobilized antibody receptors on the active GFET sensor area.

This simplifies the situation, which, as shown in Figure 4, is accomplished by providing a constant flow of a fresh analyte solution to the sensor. In a biosensing experiment, an essential quantity is the “bound fraction/ferritin”, Bf value [56], because it is proportional to the measured signal. The bound fraction is defined by the occupied number of ferritin antibodies divided by the total amount of ferritin on the active surface detection area. The antibodies bound are 0% for a ferritin sensor, and they reach 100% when the sensor surface is fully saturated with ferritin analyte molecules.

(6) $$\mathrm{Bound}\;\mathrm{fraction}=S\frac{AF}{F}\propto \;\mathrm{biosensor}\;\mathrm{signal}$$

By combining Equations (5) and (6), we can re-arrange and get the equivalent of the law-of-mass-action for active surface biosensors.

(7) $$\mathrm{Bound}\mathrm{fraction}\mathrm{in}\mathrm{equilibrium},\;B_{f}(c)=\frac{c}{c+K_{D}}$$

Equation (7) corresponds to the Langmuir isotherm [57], which is derived for the adsorption of the molecules onto surfaces (in this case, on the biosensor surface with attached antibodies) [53,58].

**Figure 5:**
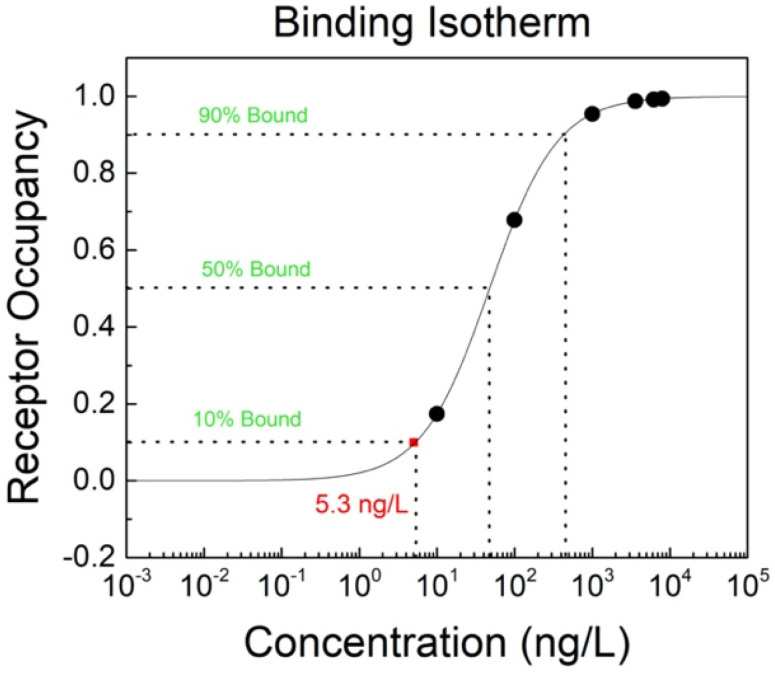
Binding isotherm for the antibody (receptor) occupancy with ferritin antigen on the GFET biosensor.

Compared to the law-of-mass-action, this method is simpler and only depends on the ferritin concentration C_F_ and the equilibrium dissociation constant for the antibodies K_D_. In the specific case of antibodies binding to ferritin antigens, the affinity constant Ka should be calculated. With the known affinity constant, the binding isotherm for the antibody occupancy with the bonded ferritin antigen can easily be plotted (Figure 5). As can be seen from the graph, the lowest ferritin antigen concentration that was successfully bonded to antibodies is equal to 5.3 ng/L (10 fM), which can be indicated as the limit of detection (LoD) for these types of developed GFET biosensors.

## 4. Conclusions

In this work, we demonstrated the possibility of using graphene to develop an FET biosensor for the detection of serum ferritin protein, whose level gives reliable information about iron deficiencies in the human body. This is the first reported GFET biosensor for ferritin detection. These GFETs were fabricated using our innovative and low-cost method of preparing a shadow mask for patterning and evaporating metal contacts on the substrate. From our analysis, the ferritin detection limit of the GFET biosensor is 5.3 ng/L (10 fM), which is the lowest detection limit reported for ferritin in the literature, while the detection range is 5.3 ng/L (10 fM) to ~0.5 µg/L (1 pM). These results show that there is excellent potential in using these GFETs for non-invasive ferritin sensing characterized by very low detection limits.

## Figures and Tables

**Table 1:** Correlating serum/plasma and salivary ferritin concentrations in healthy and iron-deficient (ID) subjects.

Normal/Healthy (µg/L)		Iron-Deficient (µg/L)		Reference
Serum/ plasma	Saliva	Serum/ plasma	Saliva	
196	6.5	-	-	[18]
225	948	169	1114	[19]
75	0.53	-	-	[20]
-	939 ± 301	-	1532 ± 466	[21]
0.30 ± 0.17	0.43 ± 0.42	0.067 ± 0.035	0.186 ± 0.085	[22]
-	169 ± 22	-	Reduced concentration compared to normal saliva [16]	[23]

**Table 2:** Ferritin-targeted micronutrient biosensors.

Sample Type	Detection Mechanism	Performance					Reference
		DL	SE	SP	R	RT	
Serum	Fluorescence	250 pM	---	---	---	---	[24]
	Fluorescence test strip	15 ng/mL	88%	97%	---	15 min	[25]
	Photonic crystal biosensors	26 ng/mL	---	---	Up to 2000 ng/mL	---	[26]
Whole blood	Lateral flow immunoassay (LFIA)	---	90%	100%	---	---	[27]
	µPAD to derive plasma; quantification via light transmission changes by a photodetector	5 ng/mL	80%	84%	Up to 50 ng/mL	15 min	[28]
PBS	Horn-like silicon nanowire FET	50 pg/mL	133.47 mV/pH	---	Up to 500 ng/mL		[29]
**PBS**	**Graphene FET**	**5.3 ng/L (10 fM)**	**---**	**---**	**Up to 500 ng/L**	**1–10 s**	**This work**

DL—detection limit; SE—sensitivity; SP—specificity; R—range; RT—response time; PBS—phosphate-buffered saline; FET—field-effect transistor.

## Supplementary Materials

The following are available online at https://www.mdpi.com/1424-8220/20/13/3688/s1, Figure S1: The IDE shadow mask pattern design specific for 4-inch wafer used in this work, Figure S2: GFET functionalization process, Figure S3: Liquid-gated FET Setup, Figure S4: Raman shift for CVD-synthesized monolayer graphene, Figure S5: Shift of I-V curve upon different stages of functionalization of another device (#7). (a) Shows initial I-V curve, as well as upon functionalization with PASE + antibody, passivation, and addition of target ferritin biomolecule of 8 ng/mL concentration. (b) Shows the change in the I-V curve upon final step, of addition of specific ferritin biomolecules Table S1: Ferritin concentration and the equivalent bound fraction.
