# Supplementary material for: Graphene-Based Biosensor for Early Detection of Iron Deficiency
Source: Sensors (Basel). 2020 Jul 1;20(13):3688. doi: 10.3390/s20133688 (PMC7374411; doi:10.3390/s20133688)
Supplement: Supplementary file 1 [file sensors-20-03688-s001.pdf]

Graphene-based biosensor for early detection of iron deficiency

Oluwadamilola Oshin [1,*], Dmitry Kireev [2,3], Hanna Hlukhova [4], Francis Idachaba [1], Deji Akinwande[2,3] and Aderemi Atayero [1]

1. Electrical and Information Engineering Department, Covenant University, Ota, Nigeria
2. Department of Electrical and Computer Engineering, The University of Texas at Austin, USA
3. Microelectronics Research Center, The University of Texas at Austin, USA
4. Institute of Complex Systems (ICS-8), Forschungszentrum Juelich, Germany

* Correspondence: damilola.adu@covenantuniversity.edu.ng

Supplementary Information

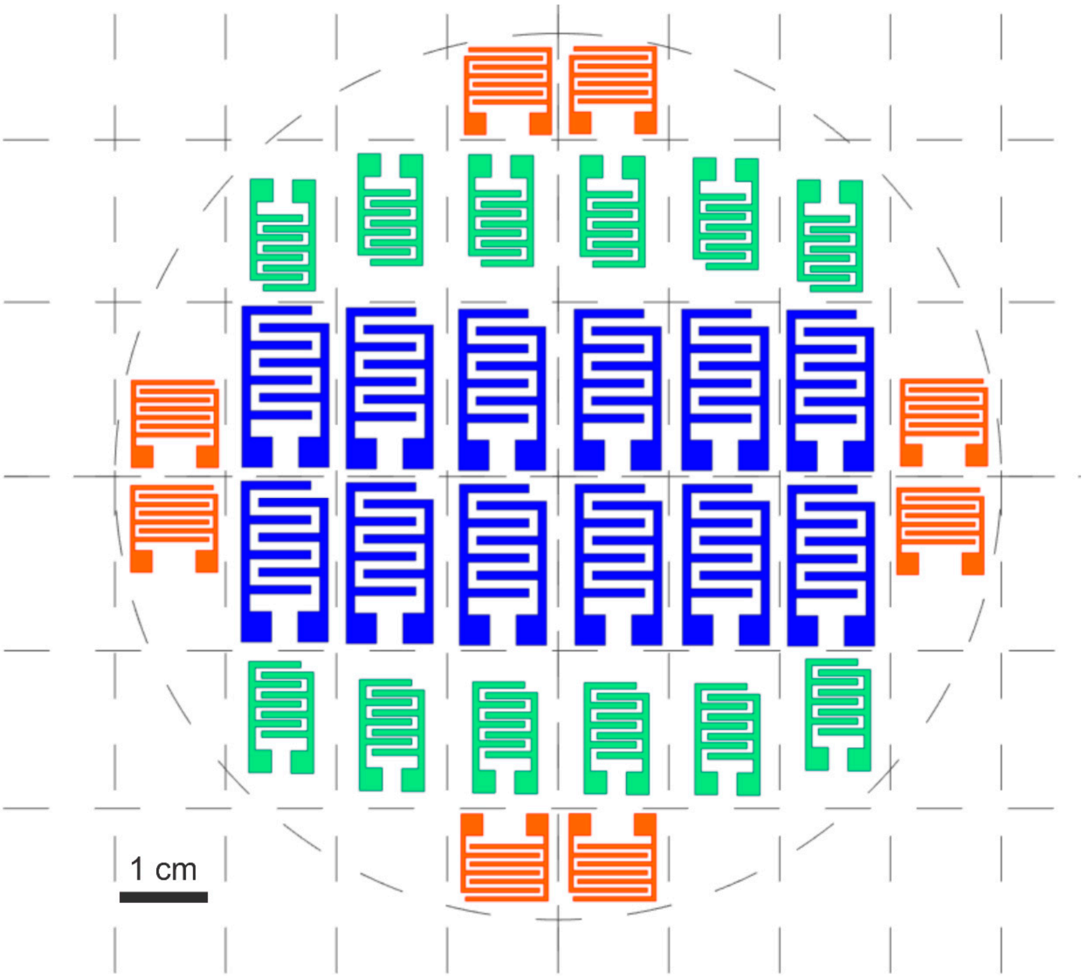

Figure S1: The IDE shadow mask pattern design specific for 4-inch wafer used in this work.

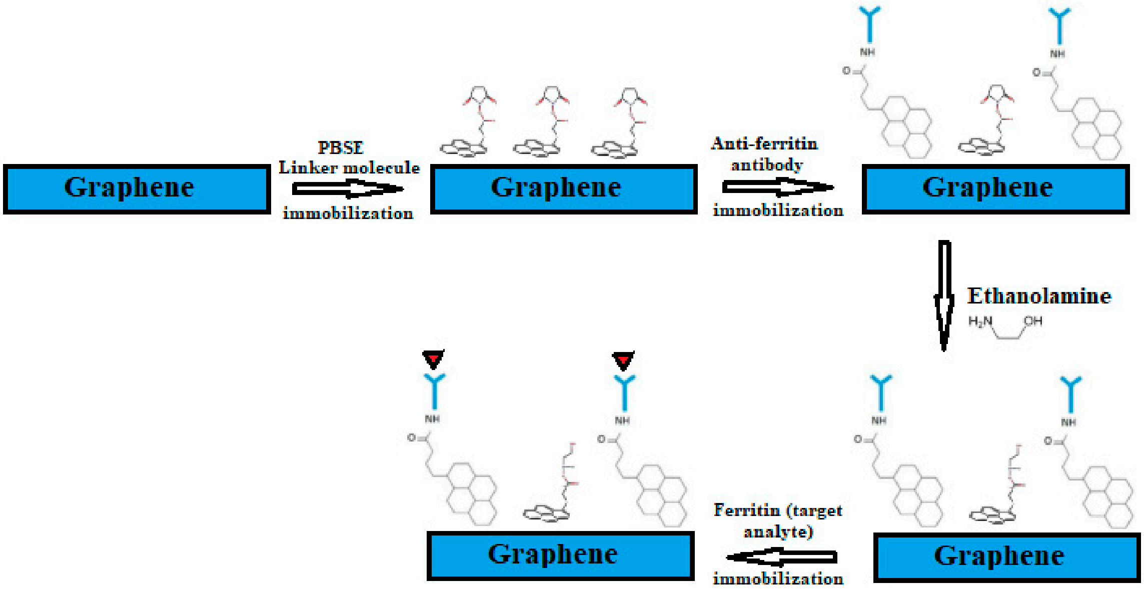

Figure S2: GFET functionalization process.

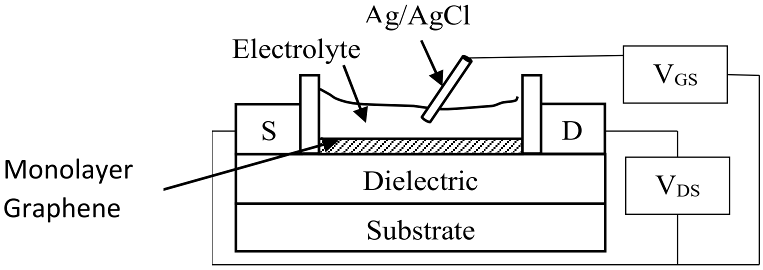

Figure S3: Liquid-gated FET Setup.

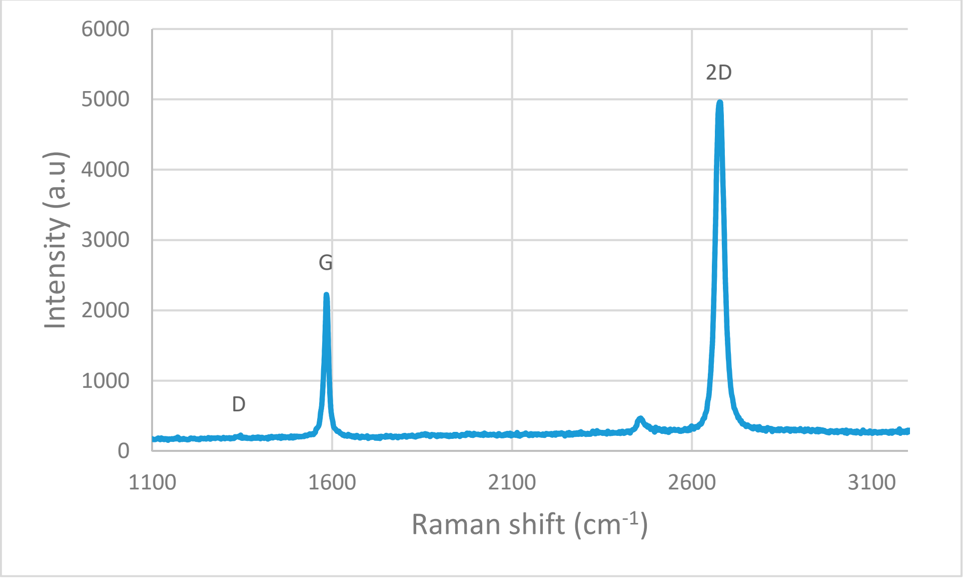

Figure S4: Raman shift for CVD-synthesized monolayer graphene.

Table S1: Ferritin concentration and the equivalent bound fraction.

Ferritin Antigen, $c_{F,n}$		Bound Fraction, B_f
10 pg/mL	21.1 fM	0.174
100 pg/mL	210.97 fM	0.678
1 ng/mL	2.11 pM	0.955
3.6 ng/mL	7.59 pM	0.987
6.18 ng/mL	13.04 pM	0.9923
8 ng/mL	16.88 pM	0.99411

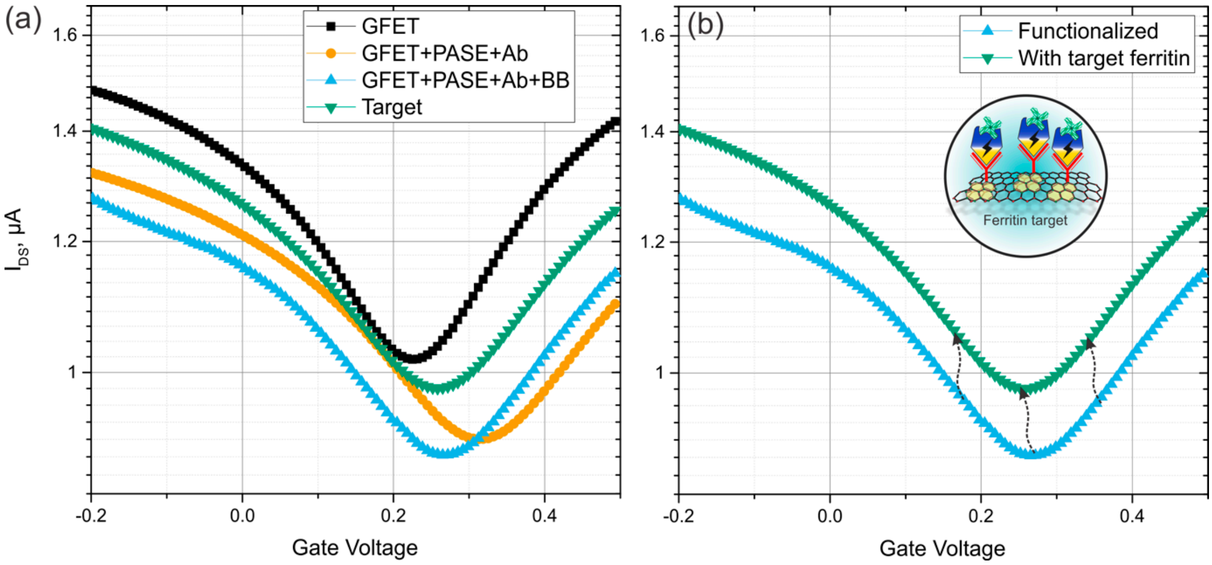

Figure S5. Shift of I-V curve upon different stages of functionalization of another device (#7). (a) Shows initial I-V curve, as well as upon functionalization with PASE + antibody, passivation, and addition of target ferritin biomolecule of 8 ng/mL concentration. (b) Shows the change in the I-V curve upon final step, of addition of specific ferritin biomolecules.
